# Pheromone cCF10 inhibits the antibiotic persistence of *Enterococcus faecalis* by modulating energy metabolism

**DOI:** 10.3389/fmicb.2024.1408701

**Published:** 2024-07-08

**Authors:** Li Zhu, Xiaobo Yang, Xinyue Fu, Panpan Yang, Xiaoli Lin, Feng Wang, Zhiqiang Shen, Jingfeng Wang, Feilong Sun, Zhigang Qiu

**Affiliations:** ^1^School of Environmental and Chemical Engineering, Xi’an Polytechnic University, Xi’an, China; ^2^Key Laboratory of Risk Assessment and Control for Environment and Food Safety, Tianjin Institute of Environmental and Operational Medicine, Tianjin, China; ^3^College of Oceanography and Ecological Science, Shanghai Ocean University, Shanghai, China; ^4^School of Public Health, North China University of Science and Technology, Tangshan, China; ^5^Key Laboratory of Karst Geological Resources and Environment, Guizhou University, Guizhou, China

**Keywords:** persister, *Enterococcus faecalis*, pheromone cCF10, biofilm, (p)ppGpp

## Abstract

**Introduction:**

Bacterial resistance presents a major challenge to both the ecological environment and human well-being, with persistence playing a key role. Multiple studies were recently undertaken to examine the factors influencing the formation of persisters and the underlying process, with a primary focus on Gram-negative bacteria and *Staphylococcus aureus* (Gram-positive bacteria). Enterococcus faecalis (*E. faecalis*) is capable of causing a variety of infectious diseases, but there have been few studies of *E. faecalis* persisters. Previous studies have shown that the sex pheromone cCF10 secreted by *E. faecalis* induces conjugative plasmid transfer. However, whether the pheromone cCF10 regulates the persistence of *E. faecalis* has not been investigated.

**Methods:**

As a result, we investigated the effect and potential molecular mechanism of pheromone cCF10 in regulating the formation of persisters in *E. faecalis* OG1RF using a persistent bacteria model.

**Results and discussion:**

The metabolically active *E. faecalis* OG1RF reached a persistence state and temporarily tolerated lethal antibiotic concentrations after 8  h of levofloxacin hydrochloride (20 mg/mL) exposure, exhibiting a persistence rate of 0.109 %. During the growth of *E. faecalis* OG1RF, biofilm formation was a critical factor contributing to antibiotic persistence, whereas 10 ng/mL cCF10 blocked persister cell formation. Notably, cCF10 mediated the antibiotic persistence of *E. faecalis* OG1RF via regulating metabolic activity rather than suppressing biofilm formation. The addition of cCF10 stimulated the Opp system and entered bacterial cells, inhibiting (p)ppGpp accumulation, thus maintaining the metabolically active state of bacteria and reducing persister cell generation. These findings offer valuable insights into the formation, as well as the control mechanism of *E. faecalis* persisters.

## 1 Introduction

The emergence of antimicrobial resistance (AMR) has resulted in increased morbidity and mortality rates, making it a serious public health concern (Li et al., 2021; Pennino et al., 2023). Worldwide, AMR is reported to be responsible for 700,000 deaths each year (Wang et al., 2019). As a result, AMR has attracted great attention, particularly regarding its formation mechanisms (Cho et al., 2018; Cepas et al., 2019; Shahin et al., 2019; Kaszab et al., 2023) and removal processes (Saha and Mukherjee, 2019; Alt et al., 2023). AMR include intrinsic (Roberts et al., 2021) and acquired resistance (Shahin et al., 2019). Intrinsic resistance is determined by chromosomes, and different bacterial cell structures and chemical compositions make them naturally insensitive to some antibacterial drugs. Acquired resistance can occur via several methods, including genetic mutations (Roberts et al., 2021) and plasmid-mediated horizontal gene transfer (Yang et al., 2023). Efforts to mitigate AMR involve various removal mechanisms, including antibiotic stewardship programs (Pallares et al., 2022), wastewater treatment (Łuczkiewicz et al., 2010) and bioremediation (Apreja et al., 2022). Generally, AMR development is attributed to the spread of antibiotic resistance genes (ARGs). Recently, however, it has been found that persisters not only promote the evolution of AMR but are also a major cause of recalcitrant infections and persistent contamination (Zhang, 2014; Gollan et al., 2019; Liu et al., 2022). Persisters, a subpopulation of bacterial cells that exist in a non-growing and non-responsive state, exhibit phenotypic but not genetic changes (Harms et al., 2016). Bacteria spontaneously enter a persistence state and could survive under various stresses, including antibiotic exposure. A biphasic kill curve revealed that persisters could survive even after exposure to lethal antibiotic concentrations (Balaban et al., 2019) and could resume normal growth after antibiotic removal, thereby recovering antibiotic sensitivity (Fang and Allison, 2023). Although their minimum inhibitory concentration (MIC) may remain constant in the dormant population, persisters could cause treatment failure and disease recurrence (Gollan et al., 2019). For example, *Staphylococcus aureus* (*S. aureus*) persisters have been associated with suppurative infection and hospital cross infection (Chang et al., 2020; Yee et al., 2022), and *Escherichia coli* (*E. coli*) persisters have been linked with recurrent urinary tract infections (Morales-Espinosa et al., 2016). Furthermore, the presence of persisters means that traditional antibiotic targets are inactive (Li et al., 2018; Bartell et al., 2020; Beam et al., 2021), limiting the accessibility of drugs (Cao et al., 2022) for bacterial infections treatment. Moreover, persistence promotes antibiotic resistance evolution (Windels et al., 2019a,b) and population tolerance development (Van den Bergh et al., 2017). Therefore, preventing persister formation is a crucial AMR control strategy.

Persistence is a defensive strategy bacteria use to resist antibiotics. Hitherto, persister cells have been found in almost all varieties of bacterial populations, including *E. coli* (Tkachenko et al., 2014; Xu et al., 2021), *Pseudomonas aeruginosa* (*P. aeruginosa*; Mlynarcik and Kolar, 2017), and *S. aureus* (Lechner et al., 2012; Kim et al., 2018), among other prevalent clinical and environmental bacteria (Kaur et al., 2018; Fernandes et al., 2022). Previous research has shown that prolonged exposure to high concentration of antibiotics (Kaur et al., 2018; Paranjape and Shashidhar, 2019), nanomaterials (Wang et al., 2022), and other conventional or innovative antimicrobial agents (Ye et al., 2022) could result in phenotypic persistence. Therefore, in the laboratory researchers used a variety of strategies to provoke persisters, including nutritional restriction, exposure to lethal concentrations of antibiotics, and so on (Svenningsen et al., 2022). These environmental stimuli triggered bacterial persister formation via influencing the membrane potential through an oxidative stress (OS)-reliant mechanism (Wang et al., 2017), regulating bacterial metabolism and toxin-antitoxin (TA; Wagner and Unoson, 2012) modules in *E. coli*. Furthermore, persister formation involves both DNA damage and repair, as well as SOS response (McCourt et al., 2014; Podlesek and Žgur Bertok, 2020). In the context of Gram-positive bacteria, it is believed that biofilms significantly impact (Liu et al., 2021; Zhang et al., 2022) persister formation in *S. aureus* and that bacterial biofilms also serve as a protective growth modality (Lebeaux et al., 2014) for acclimation to harsh environments, accounting for almost 80% (Hrynyshyn et al., 2022) of chronic infections. Moreover, Dufour et al. (2022) discovered that quorum sensing (QS) could promote bacterial persistence by regulating the TA system. Based on existing insights into these mechanisms, researchers hypothesized that specific chemicals and small molecules (Liu et al., 2021; Narimisa et al., 2021) might impede persister formation by modulating QS systems (Allegretta et al., 2017), biofilms (Lin et al., 2022; Nguyen et al., 2023), and intracellular ATP levels (Conlon et al., 2016; Aedo et al., 2019) in both Gram-positive and Gram-negative bacteria. The use of single-cell technologies including flow cytometry, microscopy, microfluidic devices, and other technologies in conjunction with each other can enhance our ability to recognize persisters (Balomenos et al., 2017; Hare et al., 2021). Currently, the mechanisms that facilitate or inhibit bacterial persistence formation are relatively constrained, with the specific target yet to be elucidated.

Currently, persister research focuses primarily on *E. coli*, *P. aeruginosa*, and Gram-positive bacteria such as *S. aureus*, whereas *E. faecalis* persisters, which frequently precipitate nosocomial infections, have received minimal attention (Van Tyne et al., 2019; Kalfopoulou and Huebner, 2020). According to research, *E. faecalis* possesses a robust cell wall (Canfield et al., 2023), making it inherently resistant (Korir et al., 2019) to various antibiotics. Moreover, it readily acquires resistance via the uptake of resistant plasmids (Tomita et al., 2023; Yang et al., 2023), transposons (Dale et al., 2015), or genetic mutations (García-Solache and Rice, 2019). Due to the severity of *E. faecalis* resistance, there are few antibiotics available for the clinical treatment of *Enterococcus* infections. Phenotypic resistance is a characteristic feature of persistent *E. faecalis* infections. Kaviar et al. (2022) discovered that *E. faecalis* clinical isolates exhibited a high proportion of persister cells and vancomycin tolerance tendency, further complicating treatment. Therefore, to control their formation and prevent chronic infections, more attention should be paid to enterococcal persisters.

Several studies have proposed strategies to combat bacterial persistence. However, favorable environmental (Wainwright et al., 2021) conditions could revive persister cells. Through single amino acid testing, Yamasaki et al. (2020) confirmed that nutrient signaling molecules could facilitate persister resuscitation. Bacteria-secreted signaling molecules could regulate bacterial behavior (Dunny and Leonard, 1997), and as an essential mode of bacterial communication, they have been demonstrated to regulate persistence processes (Zarkan et al., 2020). Specifically, it has been reported that the fatty acid signaling molecule cis-2-decenoic acid (cis-DA) can resuscitate persister cells to a metabolically active state in *E. coli* and *P. aeruginosa* (Marques et al., 2014). These findings suggest that signaling molecules could regulate bacterial metabolism. Therefore, we hypothesized that signaling molecules might sustain bacterial metabolic activity, preventing bacteria from assuming a state of persistence.

The secretion of intercellular signaling molecules among bacteria, a prevalent phenomenon, is crucially involved in bacterial behavior regulation (Mori et al., 1984; Vickerman and Mansfield, 2019). The peptide pheromones secreted by *E. faecalis* can be sensed and induced by bacteria as signal molecules. There are many similar pheromones such as cCF10, cAM373 and cAD 1 secreted by *E. faecalis* that regulate expression of conjugative plasmid transfer genes in *E. faecalis* (Mori et al., 1984; Vickerman and Mansfield, 2019). Of these, pheromone cCF10 has the highest level of attention. The amino acid sequence of the sex pheromone cCF10 is LVTLVFV (Dunny and Leonard, 1997). Pro-C is a secreted lipoprotein comprising a cleaved signal peptide from the *ccfA* gene product in *E. faecalis*. Following the initial cleavage, the released 22-amino acid signal peptide was further cleaved by Eep protease to produce C (cCF10) and released into the growth medium (Flannagan and Clewell, 2002; Chandler and Dunny, 2008; Varahan et al., 2014). Generally, cCF10 and its antagonistic signaling molecule, iCF10 (Chen et al., 2017), competitively bind to PrgX (Price et al., 2016) in OG1RF (pCF10). This process initiates *prgQ* transcription, inducing conjugative plasmid (pCF10) transfer (Chatterjee et al., 2013). Moreover, in the absence of pCF10, cCF10 can also be taken up by OG1RF through the Opp2 system (Segawa et al., 2021). However, whether the pheromone cCF10 regulates the persistence in *E. faecalis* during this process has not been investigated. Therefore, we proposed that pheromone cCF10 may prevent bacteria from transitioning to a low-energy metabolic state, impeding the formation of persisters in *E. faecalis*. Herein, we exposed *E. faecalis* to levofloxacin hydrochloride (LVF) to screen for persisters *in vitro* experiments and we investigated the impact of pheromone cCF10 on the formation of persister cells in *E. faecalis* OG1RF, along with exploring its potential molecular mechanism. Furthermore, this study aimed to enhance our understanding of the formation of *E. faecalis* persisters and provide a novel approach and theory for studying the prevention and treatment of *E. faecalis* persisters.

## 2 Materials and methods

### 2.1 Bacterial strains and culture condition

All the *E. faecalis* used herein were derived from *E. faecalis* OG1RF (ATCC 47077). The OG1RF∆*ccfA* strain was constructed in our lab as outlined in Yang et al. (2023). Supplementary Text S1 presents the details. The bacteria were grown in the brain heart infusion (BHI) medium (Coolaber Science and Technology, China) at 37°C and 150 rpm agitation.

### 2.2 The biphasic killing curve experiment of *Enterococcus faecalis*

First, OG1RF cultures in the logarithmic growth phase were initiated by inoculating them into the BHI medium (1:1,000). Following that, cells were grown for 4 h and then exposed to different LVF concentrations. Herein, LVF concentrations were MIC-based. Supplementary Text S2 describes antibiotic type selection and MIC measurement. Bacteria were treated with various antibiotic concentrations, followed by sample collection at different time intervals for enumeration. Subsequently, the cultures were gradient-diluted with a Phosphate-Buffered Solution (PBS) and then grown on BHI agar for counting.

### 2.3 Detection of the persistence rate during the growth of *Enterococcus faecalis*

First, OG1RF cultures in the logarithmic growth phase were inoculated into the BHI medium (1:1000) and then cultured at 37°C. At specific culture time points (4, 4.5, 5, 5.5, and 6 h), a sample was removed from the culture medium and challenged with LVF at a final concentration of 20 mg/L. The mixed cultures were then incubated at 37°C for 8 h. Subsequently, the samples were diluted with PBS before inoculating their appropriate dilutions on BHI agar to count the surviving bacteria. Simultaneously, the samples’ bacterial concentrations before LVF treatment was determined. The results were presented as a colony-forming unit (CFU)/mL, and the persister frequency *(*f*)* was determined using the following Formula (1):


(1) $$f=\frac{N_{2}}{N_{1}}$$

where $N_{2}$ represents the number of bacteria in the culture after antibiotic treatment (CFU/mL), and $N_{1}$ is the number of bacteria in the culture before antibiotic treatment (CFU/mL).

### 2.4 Effects of cCF10 on persister formation in *Enterococcus faecalis*

First, OG1RF cultures in the logarithmic growth phase were inoculated into the BHI medium (1:1,000) and then supplemented with different concentrations of the pheromone cCF10 (8, 10, 12, 14, 16, and 20 ng/mL). After incubating the cultures for 3 h, the bacteria were collected through centrifugation at 8,000 rpm for 3 min. The collected material was re-suspended in the BHI medium, and then exposed with LVF at a final concentration of 20 mg/L before determining the persistence rate as earlier mentioned. We detected the persistence rate of OG1RF∆*ccfA* to further verify the effect of cCF10 on the formation of *E. faecalis* persistence. We also examined the effects of different cCF10 concentrations (1, 10, and 20 ng/mL) on the persistence rate of OG1RF∆*ccfA.* Notably, Kingsray Biotechnology Co., Ltd. (Nanjing, China) synthesized the peptide pheromone used herein, the 7-amino acid sex pheromone cCF10 (C-clumping-inducing peptide, amino acid sequence LVTLVFV; Dunny and Leonard, 1997). To confirm the specificity of cCF10, a site-mutated sequence called cCF10-F (sequence = LVFLVTV) was employed and cCF10-F were synthesized by GenScript (China). The procured pheromones cCF10 and cCF10-F were dissolved in acetonitrile (Maclean’s, China), respectively, and then stored at −20°C in the dark.

### 2.5 Extraction and detection of the extracellular pheromone cCF10

We extracted the extracellular pheromone per the methodology described by Zhou et al. (2023). Briefly, the collected samples were placed on ice for 20 min and then centrifuged at 6,000 rpm for 10 min at 4°C. The supernatant was then filtered using a 0.22 μm filter (SLGP033RB Millipore, Massachusetts, United States) and mixed with ammonium hydroxide solution and acetonitrile (both from Shanghai Macklin Biochemical Technology Co. Ltd., China) at a ratio of 8:1:1. The resulting mixtures were vortexed at 1,400 rpm for 15 min at room temperature (RT) and then centrifuged for 15 min at 25,000 rpm (ST16R; ThermoFisher Scientific, Massachusetts, United States). Following that, thorough mixing was done with the supernatant and an equivalent amount of an aqueous solution of 10% ammonium hydroxide. The cCF10 elution process was as follows. First, the extraction column (Sep-Pak C18 WAT054945 Waters, Massachusetts, United States) was activated by sequentially flowing 5 mL acetonitrile through it, followed by 5 mL water at a rate of one drop/s. A volume of 30 mL of test solution was passed through the extraction column at a speed of one drop per 3 s. Subsequently, to eliminate water-soluble impurities from the column bed matrix material within the extraction column or those that have been absorbed during activation or sample loading, 2 mL of water was introduced as an eluent. This step was followed by flushing with an aqueous acetonitrile solution (30%), also amounting to 2 mL, to ensure the impurities were properly removed. Finally, the collected eluate was sent to Science Compass (Zhejiang, China) for Liquid Chromatography Mass Spectrometry (LCMS) testing. Supplementary Text S4 presents the detailed detection procedure. Preparation of Scanning Electron Microscope (SEM) samples.

The cell pellet was gently re-suspended in a pre-cooled 2.5% glutaraldehyde fixative for 24 h at 4°C. The fixative was then removed from the sample via centrifugation at 6000 rpm for 5 min, followed by dehydration using an ethanol gradient approach. Ultimately, the bacteria underwent lyophilization with a FDU1200 EYELA freeze-dryer (Tokyo, Japan) and were then imaged using a Sigma 300 SEM (Zeiss, Germany).

### 2.6 Transcriptome sequencing

Firstly, *E. faecalis* OG1RF normal bacteria, persisters, and cCF10 treatment persisters were collected as previously described. The bacterial cultures were first collected via centrifugation at 8,000 rpm for 3 min. The collected residues were then cooled in liquid nitrogen for 15 min and stored at −80°C before being sent to Allwegene Technology Co. Ltd. (Beijing, China) for transcriptome sequencing. Supplementary Text S5 describes the specific sequencing methods employed. The transcriptome data (project number PRJCA026007) has been deposited in the China National center for Bioinformation. The URL is https://ngdc.cncb.ac.cn/bioproject/browse/PRJCA026007.

### 2.7 Measurement of biofilm formation

The OG1RF strain was cultured in a six-well plate, and any unadhered bacteria were gently washed off with PBS. The plates with adherent biofilms were then air-dried at RT and fixed with methanol (1 mL/well) for 15 min. The methanol was discarded, and the plates were air-dried again. A 1% crystal violet staining solution (1 mL/well) was then added to stain the biofilms for 1 h. Subsequently, the PBS was used to carefully wash away the crystal violet staining solution until it became colorless, after which the plates were left to air dry. The OD_570_ was then determined using a Multifunctional Fluorescent Enzyme Labeler (Spectra Max M5, United States).

### 2.8 Adenosine triphosphate measurement

First, the bacterial cultures were centrifugally washed with PBS at 6,000 rpm for 5 min and then subjected to ATP measurement per the instructions in the ATP Assay Kit (Beyotime Biotechnology Co. Ltd., China). Chemiluminescence measurements were recorded using MFEL (SpectraMax M5, United States), and the data were normalized to the total amount of protein measured per the instructions in the BCA Protein Concentration Measurement Kit (Beyotime Biotechnology Co. Ltd., China).

### 2.9 Total RNA extraction and real-time fluorescent quantitative reverse transcription polymerase chain reaction analysis

The samples were transferred to ice for immediate cooling, and total RNA extraction was performed per the instructions in the Gram-Positive Microbes RNA Isolation Kit (Beibei Biotechnology Co, China). Subsequently, reverse transcription polymerase chain reaction (RT-qPCR) was conducted to convert total RNA into cDNA using the first strand cDNA synthesis kit (Tiangen, China) and random primers. Subsequently, the Power Up SYBR Master Mix (ThermoFisher, United States) was used for real-time PCR analysis, along with the CFX96 Real-Time System (Bio-Rad Laboratories Inc., Hercules, United States). The absolute quantification method was utilized for determining the mRNA levels of specific genes related to pheromones, such as those encoding pheromones and responding to them. The 16S rRNA gene was utilized as an internal reference for normalization purposes. The primer sequences for RT-qPCR analysis can be found in Supplementary Table S1, and were designed with the assistance of DNASTAR.

### 2.10 Statistical analysis

The experiments were independently repeated a minimum of three times. Data analysis was conducted with SPSS 25 software (IBM, Armonk, NY). All data were presented as mean ± standard deviation (SD) and were analyzed using the independent samples t-test or one-way analysis of variance followed by the student–Newman–Keuls test. Results or differences with *p* < 0.05 were considered statistically significant.

## 3 Results

### 3.1 Pheromone cCF10 prevented the formation of persister cells in *Enterococcus faecalis*

Herein, we first established a persistent bacteria model and subjected *E. faecalis* strains to a series of persister analyses to examine the effect of pheromone cCF10 on the formation of *E. faecalis* persister cells (Supplementary Figures S1, S2). According to the “biphasic killing” curve, bacterial populations decreased sharply after antibiotic exposure and plateaued at 8 h (Supplementary Figure S2B). Furthermore, the number of surviving bacteria was almost stable when antibiotic concentrations exceeded 20 mg/L, indicating that the surviving bacteria were persisters (Balaban et al., 2004; Lewis, 2007; Kint et al., 2012; Harms et al., 2016). Based on SEM images (Supplementary Figure S2C), normal-growing *E. faecalis* cells exhibited a relatively round and full morphology, whereas LVF-exposed cells shriveled, with a large amount of biofilm attached to their surfaces, further indicating that the surviving bacteria were persisters. Extracellular polymers (EPS) is mainly a number of polymer substances, such as polysaccharides, proteins and nucleic acids. Our results showed that more polysaccharides, proteins and nucleic acids were produced around the persisters compared to normal bacteria (Supplementary Figure S3). Unlike dead bacteria, the persistent bacteria were able to resuscitate after the removal of antibiotic pressure. To illustrate this point, we added electron microscope images of the persistent bacteria at different resuscitation times (Supplementary Figure S4). We observed changes in the morphology of bacteria that had been screened for antibiotics when added to fresh media. We found that after 1 h of resuscitation, most of the bacteria in the visual field returned to normal bacterial form. In addition, we also compared the resuscitation curves of the persisters and the sterilization curves of the recovered persisters. We obtained the same growth curve as normal bacteria (Supplementary Figure S5A). Then we exposed the resuscitated to 20 mg/L of levofloxacin hydrochloride for 12 h. We found that the screened bacteria were still sensitive to levofloxacin hydrochloride (Supplementary Figure S5B). Our results indicated that the bacteria we screened were persisters rather than resistant bacteria.

**Figure 1:**
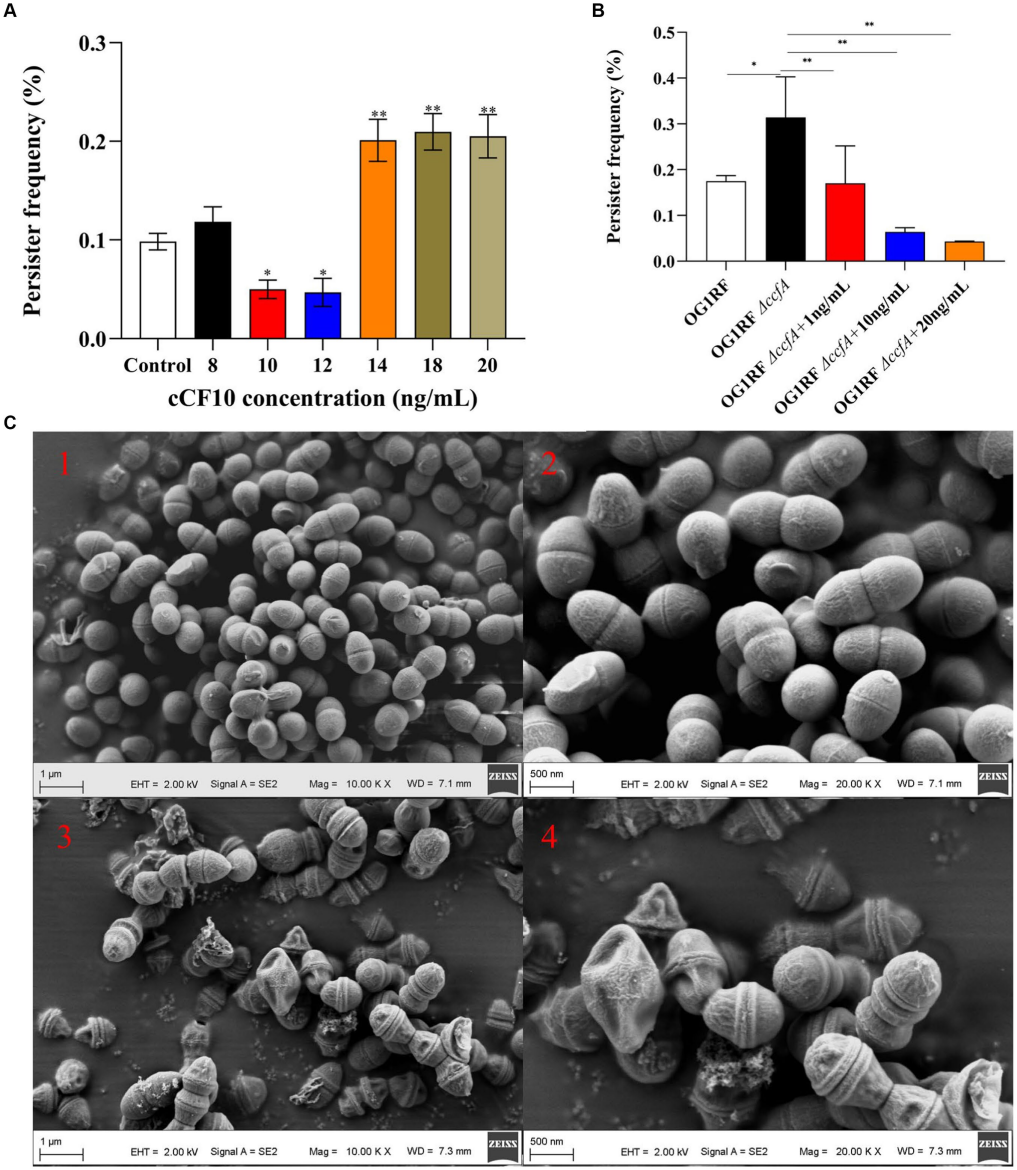
Pheromone cCF10 inhibited the formation of persister cells. **(A)** The effects of different concentrations of cCF10 on persister rate in OG1RF. **(B)** Influence of different concentrations of cCF10 on persister rate in OG1RF*∆ccfA*. The results represent the mean ± standard deviation (SD) of three biological replicates. **(C)** Representative SEM images of showing the morphology of bacteria after LVF treatment in the OG1RF*∆ccfA* (1–2) and the control group (3–4). Significant differences between groups are presented with ^*^p < 0.05 and ^**^p < 0.01.

Although bacterial growth was not affected when *E. faecalis* OGIRF was exposed to 10 and 12 ng/mL cCF10 concentrations (Supplementary Figure S6), the persister rate decreased from 0.109 to 0.050 and 0.047% (Figure 1A). This finding indicated that cCF10 could inhibit the formation of *E. faecalis* persisters. Interestingly, the persister rate of *E. faecalis* increased to 0.201%, 0.211%, and 0.205% when pheromone concentrations reached 14, 18, and 20 ng/mL, respectively. It could be attributed to the fact that the excessively high pheromone concentrations exceeded the regulatory range of *E. faecalis*, interfering with the physiological metabolism of the bacterium.

We also detected the persister rate of a mutant OG1RF*∆ccfA* strain to verify the effect of cCF10 on the formation of *E. faecalis* persistence. According to the results, the persister rate of the mutant strain increased to 0.31%. On the other hand, adding exogenous cCF10 at 10 and 20 ng/mL concentrations reduced the persister rate to 0.064% and 0.043%, respectively (Figure 1B), further indicating that the pheromone cCF10 could inhibit the persistence of *E. faecalis* within a certain range. To confirm the specificity of cCF10, we employed a site-mutated sequence called cCF10-F. Our results indicated that unlike cCF10, cCF10-F did not impact the formation of OG1RF persisters (Supplementary Figure S7). Based on SEM images (Figure 1C), normal-growing OG1RF∆*ccfA* cells exhibited a rounded and plump morphology, whereas LVF-exposed cells had a shriveled appearance, indicating that the surviving bacteria were persisters.

### 3.2 Accumulation of biofilm played a crucial role in the generation of *Enterococcus faecalis* persisters

**Figure 2:**
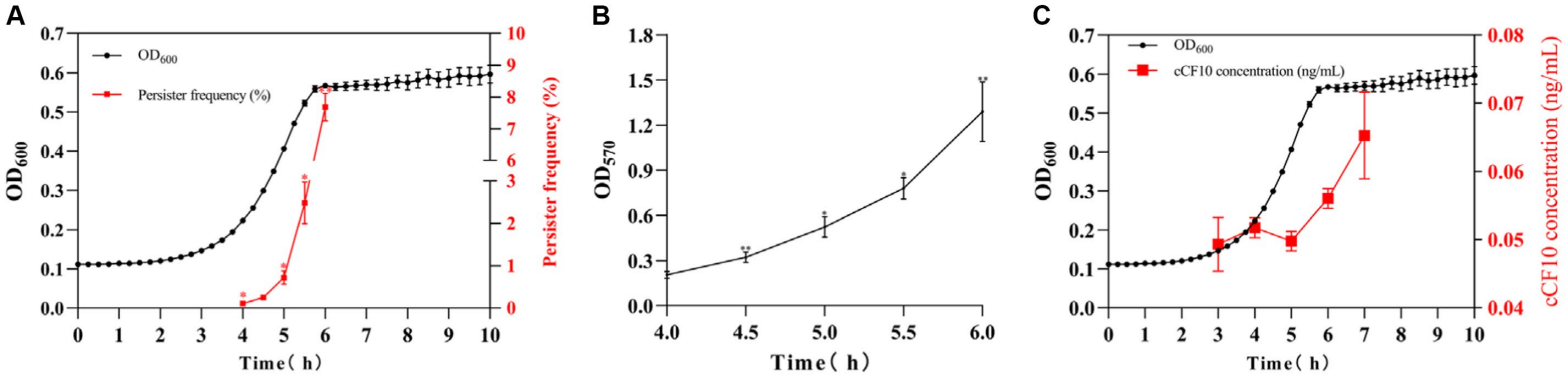
The formation of the persistence depends on biofilm accumulation in *Enterococcus faecalis*. Changes in persister rates **(A)**, biofilm accumulation **(B)**, and cCF10 concentration **(C)** at different growth periods in OG1RF. Significant differences between groups were calculated using repeated measures variance and are expressed as ^*^p < 0.05 and ^**^p < 0.01. The results represent the mean ± SD of three biological replicates.

Herein, the persisters were screened with 20 mg/L LVF at different culture times to evaluate the changes in persister rate during the OG1RF growth stages. The bacteria reached the logarithmic phase at 4 h, at which the persister rate was 0.11%, and plateaued at 6 h at which the persister rate was 7.68%. Furthermore, the persister generation rate gradually increased with the growth of OG1RF, exhibiting 0.25%, 0.72%, and 2.48% persister rates after growth for 4.5, 5, and 5.5 h, respectively (Figure 2A). To explore the potential mechanisms, we evaluated the biofilm content of OG1RF during the growth process. The results (Figure 2B) revealed OG1RF biofilm accumulation during the growth process (the OD_570_ changed from 0.204 to 1.289). Notably, although there was a concomitant increase in pheromone content as the bacterial population proliferated (Figure 2C), this heightened pheromone concentration did not cause a decline in the persister rate. Furthermore, the biofilm exerted its primary influence during this period. Additionally, we measured biofilm content in *E. faecalis* to investigate the potential impact of pheromones on biofilm formation. According to the results, bacteria accumulated a large amount of biofilm after persistence (OD_570_ increased from 0.442 to 0.600 to 0.601), and cCF10 addition did not alter biofilm accumulation (Supplementary Figure S8). To support this idea, we added the results of different concentrations (8, 10, 12, 14, 18, and 20 ng/mL) of cCF10 on OG1RF biofilm formation. Our results suggested that pheromones do not affect the formation of bacterial biofilms compared to control group (Supplementary Figure S9).

### 3.3 Gene regulatory pathways involved in cCF10 inhibited *Enterococcus faecalis* persistence

**Figure 3:**
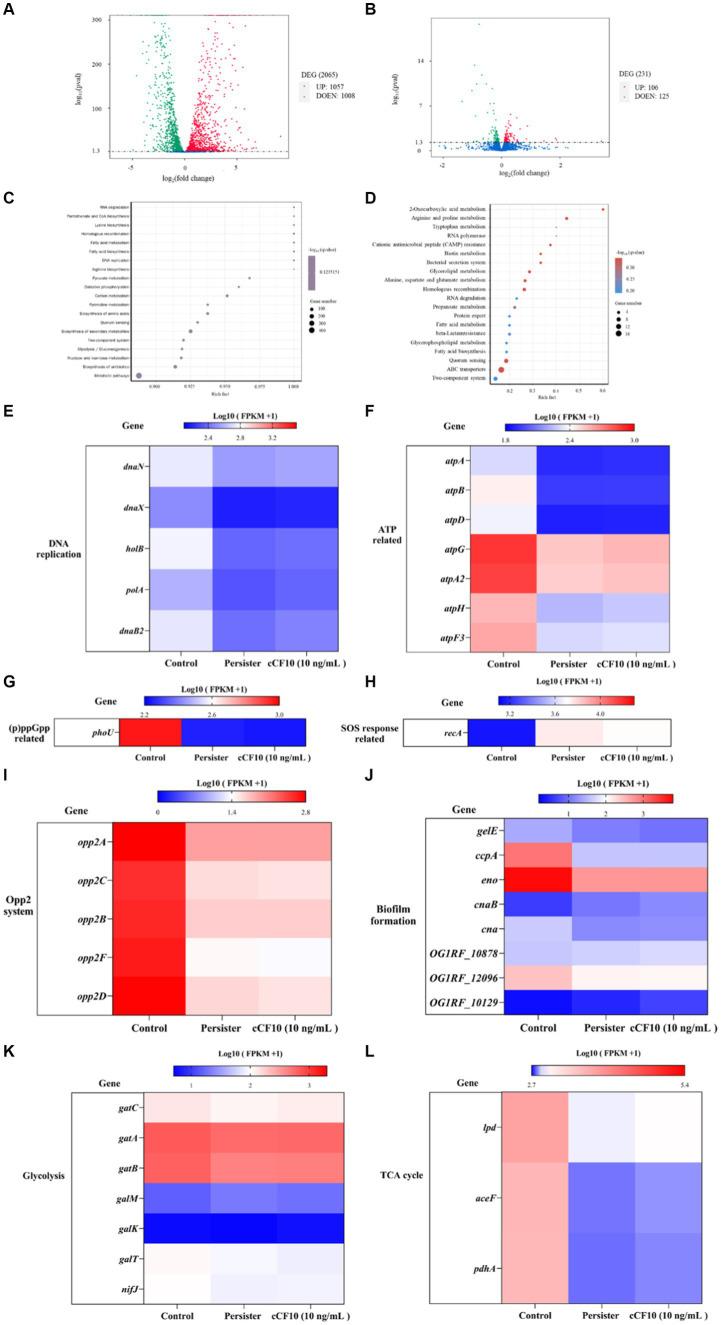
Alterations in the abundance of key genes associated with the persistence. **(A)** A volcano map of differential genes in the normal bacteria and persister cells. DEG, differentially expressed genes. **(B)** A volcano map of differential genes in the persister cells and 10 ng/mL cCF10 treatment groups. Red dots represent up regulation, and green dots indicate down-regulated. KEGG pathway enriched analysis. **(C)** The enriched pathway involved in target genes were showed in the normal bacteria and persister cells. **(D)** The enriched pathway involved in target genes were showed in the persister cells and 10 ng/mL cCF10 treatment groups. KEGG, Kyoto Encyclopedia of Genes and Genomes. Rich factor refers to the ratio of the number of differentially expressed genes enriched to the number of annotated genes in the pathway. Q-value (value range 0–1) is the *p*-value after correction by multiple hypothesis testing. Changes in the expression of genes related to DNA replication **(E)**, ATP **(F)**, (p)ppGpp **(G)**, SOS response **(H)**, Opp2 system **(I)**, biofilm formation **(J)**, glycolysis **(K)** and TCA cycle **(L)** in different groups (*N* = 4). TCA, tricarboxylic acid cycle.

Herein, we examined transcript level changes to further determine the molecular mechanisms of *E. faecalis* persistence and the mechanism by which cCF10 inhibits OG1RF persistence. We described the process of transcriptomics analysis in detail in Supplementary Text S5. Persisters had 2,065 differential genes compared to normal bacteria (Figure 3A). On the other hand, the cCF10 treatment group had 231 differential genes compared to the persisters group (Figure 3B). The top 20 enriched pathways are shown in order of q-value from smallest to largest. DEGs were mainly concentrated in metabolic pathways in normal and persisters groups (Figure 3C). On the other hand, in the persisters and cCF10 treatment groups, the results showed that the DEGs were mainly enriched in ABC transfer system (Figure 3D). Furthermore, in the persisters group, the expression levels of genes involved in DNA replication (Figure 3E) and ATP synthesis (Figure 3F) were decreased compared to normal bacteria. Among genes involved in DNA replication, *danB2* exhibited the most significant changes (Figure 3E), with reductions to 0.89 and 0.90 times that of the control group in the persister and cCF10 treatment groups, respectively. On the other hand, among the genes associated with ATP synthesis, *atpB* (Figure 3F) had the most significant alterations, with reductions to 0.79-fold and 0.80-fold that of the control group in the persister and cCF10 treatment groups, respectively. In addition, to further verify whether cCF10 could alter the energy metabolism of OG1RF and prevent bacterial persistence, we focused on changes in gene expression levels associated with glycolysis and tricarboxylic acid (TCA) cycling. Clearly, the expression levels of genes related to metabolism decreased after the bacteria entered the persistence state. In cCF10 treatment group, the expression levels of genes (*gatC, gatA*, *gatB*, *galK*, *nifJ*, *lpd*, *aceF*, and *pdhA*) were all increased. Of these, *galk* showed the most significant change with a 1.07-fold increase compared to the persisters group (Figures 3K,L). Furthermore, *phoU* expression (Figure 3G) in the persister and cCF10 treatment groups were 0.89-fold and 0.90-fold that of the control group, respectively. These findings indicate a notable *phoU* downregulation following bacterial persistence. Furthermore, *recA*, which is associated with SOS response, was upregulated by 1.19-fold (in the persisters group) and 1.17-fold (in the cCF10 treatment group) relative to the control group (Figure 3H). The Opp2 system serves as the conduit for cCF10 secretion and uptake in OG1RF (Segawa et al., 2021). In the persisters group, the expression levels of genes involved in Opp2 system were decreased compared to normal bacteria (Figure 3I). However, no changes in Opp2 system gene expression were found in cCF10 treatment group compared to persisters (Figure 3I). In order to further verify this result, we carried out laboratory verification in 3.4. Our results have shown that the accumulation of biofilm played a crucial role in the generation of *E. faecalis* persisters. Although transcriptomic results showed that the expression of several genes (*gelE*, *ccpA*, *eno*, *can* and *OG1RF_12096*) associated with biofilm formation was mostly reduced in the persistent bacteria compared with normal bacteria (Figure 3J). However, no changes in biofilm formation genes expression were found in cCF10 treatment group compared to persisters. In addition, we added different concentrations of cCF10 during the growth of OG1RF in order to further verify the effect of cCF10 on OG1RF biofilm formation. Consistently, the addition of cCF10 did not alter the formation of OG1RF biofilms (Supplementary Figure S9).

### 3.4 cCF10 prevented bacterial persistence by motivating the Opp2 system and hampering (p)ppGpp accumulation

**Figure 4:**
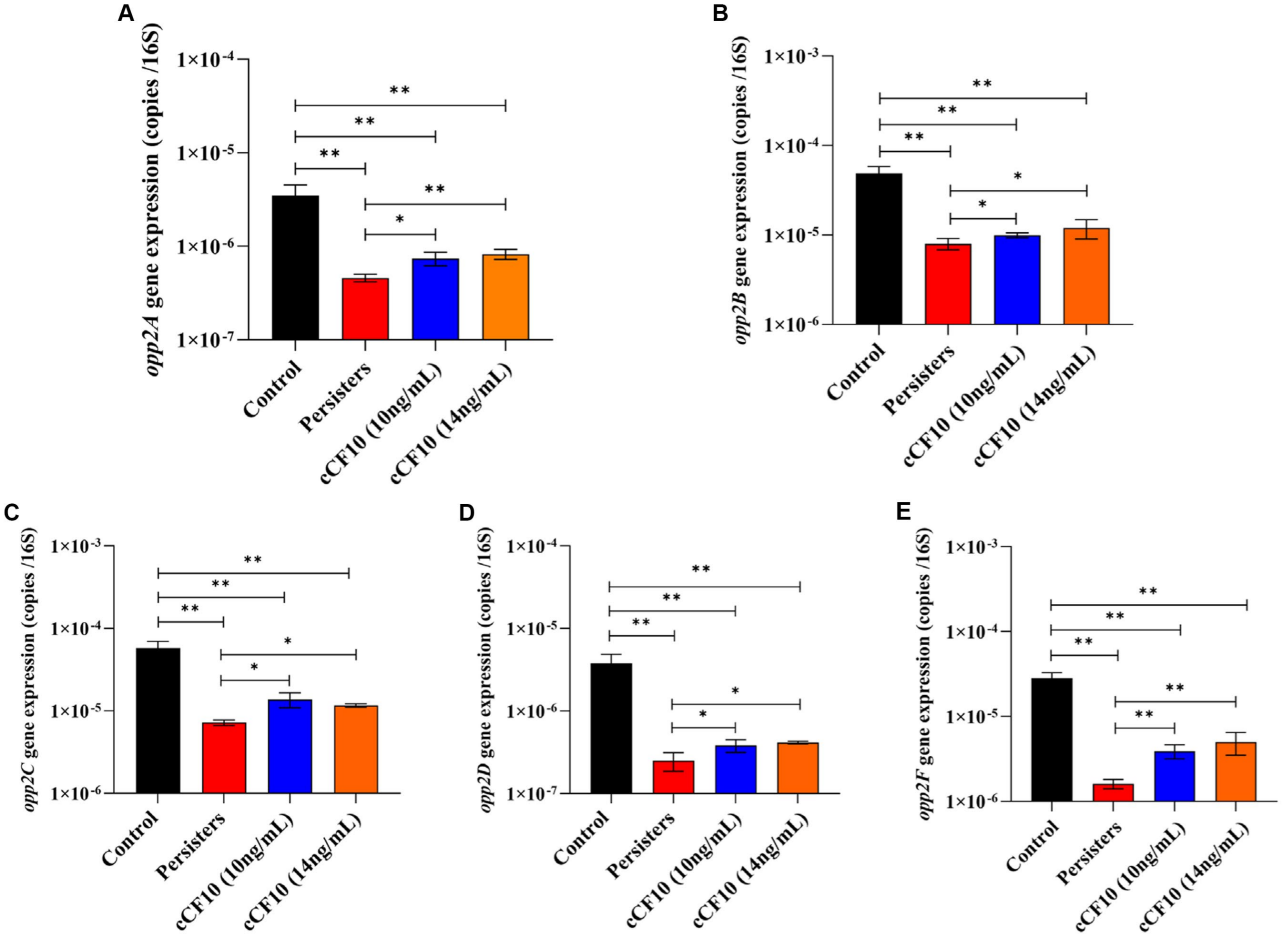
Exogenous cCF10 induces Opp system and enters cells in *Enterococcus faecalis*. Expression of the related genes of Opp system including *opp2A* **(A)***, opp2B* **(B)***, opp2C* **(C)***, opp2D* **(D)**, and *opp2F* **(E)** in different treatment groups. Significant differences between groups were calculated using analysis of variance and presented by ^*^p < 0.05 and ^**^p < 0.01. The results represent the mean ± SD of three biological replicates.

**Figure 5:**
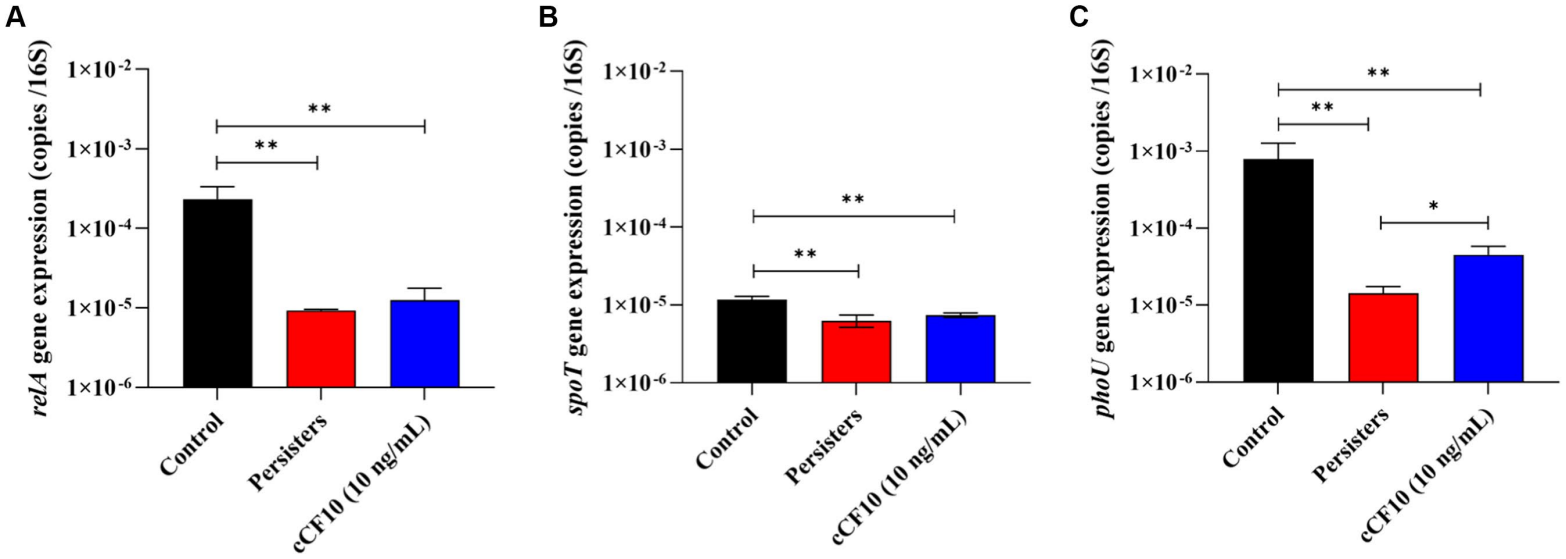
cCF10 inhibits (p)ppGpp accumulation. The expression of the (p)ppGpp encoding genes *relA* **(A)** and s*poT* **(B)** in different treatment groups. The expression of *phoU*, a gene encoding the regulatory protein PhoU of the phosphate transport system, in different treatment groups **(C)**. Significant differences between groups were obtained using analysis of variance and presented with ^*^p < 0.05 and **p < 0.01. The results represent the mean ± SD of three biological replicates.

The Opp system serves as the conduit for pheromone secretion and uptake (Segawa et al., 2021). Segawa et al. (2021) discovered that pheromone uptake in *E. faecalis* OG1RF was primarily dependent on the Opp2 system. The OppA lipoprotein is a well-conserved protein known for its ability to bind peptides. It interacts with an ABC transporter system consisting of two channel-forming proteins (OppB and OppC) and two membrane-associated ATPases (OppD and OppF; Segawa et al., 2021). Herein, we found that the expression of genes related to the Opp2 system decreased after bacteria entered the persistence phase (Figure 4), indicating that the pheromone-binding ability of *E. faecalis* persisters was reduced and that the uptake pathway was partially blocked. However, *opp2A* expression (Figure 4A) increased by 1.61-fold (10 ng/mL of cCF10) and 1.11-fold (14 ng/mL of cCF10) relative to the persisters group, suggesting that the cCF10-binding ability was improved. Similar changes were observed in *opp2B*, *opp2C, opp2D*, and *opp2F*. The expression of *opp2B* (Figure 4B) increased, respectively, by 1.25-fold (10 ng/mL of cCF10) and 1.50-fold (14 ng/mL of cCF10) relative to the persisters group, suggesting that the cCF10-binding ability was improved. The expression of *opp2C* (Figure 4C) increased, respectively, by 1.90-fold (10 ng/mL of cCF10) and 1.62-fold (14 ng/mL of cCF10) relative to the persisters group, suggesting that the cCF10-binding ability was improved. The expression of *opp2D* (Figure 4D) increased, respectively, by 1.53-fold (10 ng/mL of cCF10) and 1.66-fold (14 ng/mL of cCF10) relative to the persisters group, suggesting that the cCF10-binding ability was improved. The expression of *opp2F* (Figure 4E) increased, respectively, by 2.43-fold (10 ng/mL of cCF10) and 3.11-fold (14 ng/mL of cCF10) relative to the persisters group, suggesting that the cCF10-binding ability was improved. These findings suggested that cCF10 could induce the Opp2 system and enter bacterial cells.

(p)ppGpp is a signaling molecule that bacteria produce when they encounter challenging conditions, such as exposure to antibiotics. The build-up of (p)ppGpp can ultimately interfere with the movement of protons and inhibit ATP synthesis, leading to bacterial dormancy (Shang et al., 2020). Alterations in (p)ppGpp production negatively affect bacterial stress survival and virulence in *E. faecalis*, and (p)ppGpp directly inhibits the activity of enzymes involved in GTP biosynthesis (Gaca et al., 2013, 2015). According to research, RelA (synthase) and SpoT (hydrolase) maintain a steady state of (p)ppGpp (Hobbs and Boraston, 2019). At the same time, *phoU* was reported to affect the intracellular level of (p)ppGpp (Shang et al., 2020). Herein, *relA* (Figure 5A) and *spoT* (Figure 5B) expression decreased after the formation of persisters, and no significant changes were observed in the cCF10 treatment group. Furthermore, *phoU* expression (Figure 5C) in the cCF10 treatment group was 4.5 × 10^−5^ copies/16 s RNA, a 3.21-fold increase compared to the persisters group. These findings indicate that cCF10 could inhibit persistence by suppressing (p)ppGpp production in *E. faecalis*.

### 3.5 cCF10 reduces persister cell generation via maintaining the metabolically active state of bacteria

**Figure 6:**
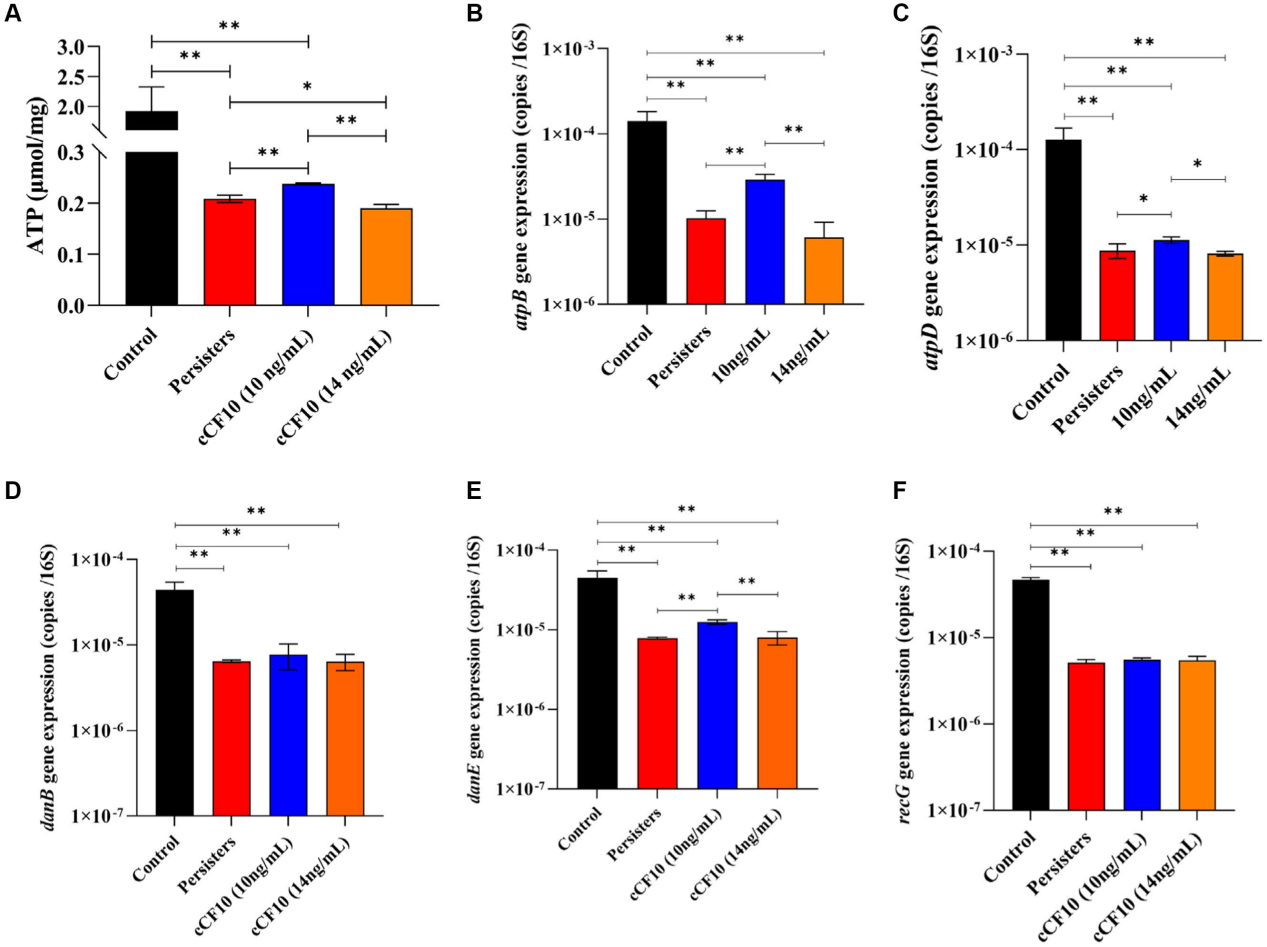
cCF10 maintains the metabolically active state of *Enterococcus faecalis*. Changes in ATP synthesis in the indicated treatment groups **(A)**. The expression of the ATP related genes *atpB* **(B)** and *atpD* **(C)** in different groups. The expression of *danB* **(D)***, danE* **(E)**, and *recG* **(F)** genes related to DNA replication in the indicated groups. Significant differences between groups were analyzed using analysis of variance and are presented with ^*^p < 0.05 and ^**^p < 0.01. The results represent the mean ± SD of three biological replicates.

Repressed metabolic activity is a distinctive feature of bacterial persistence (Amato et al., 2014). Herein, When OG1RF entered the persistence state, ATP level decreased from 1.91 μmol/ mg protein to 0.20 μmol/ mg protein. We also observed that compared to the persisters group, 10 ng/mL cCF10 treatment group significantly promoted ATP synthesis (about 1.16-fold). However, the ATP content decreased by ~0.91 times relative to that of the persisters group after adding 14 ng/mL of cCF10 (Figure 6A). Additionally, we assessed the expression levels of genes involved in energy metabolism, including ATP synthesis and DNA replication to further investigate the effect of cCF10 in preventing the formation of persister cells. As expected, *atpB* (Figure 6B) and *atpD* (Figure 6C) were downregulated after persistence (~0.073-fold and 0.069-fold of normal bacteria, respectively). After cCF10 exposure, *atpB* and *atpD* were upregulated by 2.82-fold and 1.29-fold, respectively, compared to the persisters group, further implying that cCF10 could inhibit persistence by increasing ATP levels. Similarly, *danB* (Figure 6D), *danE* (Figure 6E), and *recG* (Figure 6F) expression levels in persister cells were 0.15, 0.18, and 0.11 times those in normal bacteria, respectively. Notably, cCF10 upregulated *danE* (Figure 6E) from 7.9 × 10^−6^ to 1.3 × 10^−5^ copies/16 s RNA (~1.6-fold increase) compared to persisters. These findings illustrated that cCF10 reduces the generation of persister cells by maintaining the metabolic activity of *E. faecalis* OG1RF.

## 4 Discussion

Persisters are key in the recurrence of chronic infections (Harms et al., 2016), exerting a significant influence on disease persistence and treatment outcomes. Persister cells have been the focus of extensive research in recent times. Persisters, specifically *Enterococci*, the second most Gram-positive pathogen (Kalfopoulou and Huebner, 2020), are a common cause of chronic recurrent infections such as chronic endocarditis. Although *Enterococci*-initiated nosocomial infections may potentially be associated with bacterial persistence (Conlon, 2014), there are few studies on *Enterococci* persisters. Herein, we investigated the processes and mechanisms underlying the formation of *E. faecalis* persistence using an *E. faecalis* persistence model. The persistence rate of *E. faecalis* OG1RF was as high as 0.109%, ~10-fold greater than that of Gram-negative bacteria such as *E. coli* (Wang et al., 2022). Our findings indicated that *Enterococci* exhibited a heightened propensity for persister cell formation, warranting widespread attention. According to research, *Enterococci* biofilms exhibit inherent antimicrobial tolerance, posing a significant challenge to managing infections (Ch’ng et al., 2019) and potentially contributing to persistence. This study revealed that biofilm accumulation promoted persister formation in *E. faecalis* (Figure 2). Consistently, Kaviar et al. (2022) reported that the expression of biofilm-associated genes (*esp., agg*, and *gelE*) were higher among persisters compared to non-persister *E. faecalis* isolates. Therefore, biofilm enrichment serves as a pivotal step in *E. faecalis* persistence formation.

Numerous studies have proposed strategies for combating bacterial resistance (Allegretta et al., 2017; Baldry et al., 2020; Liu et al., 2020, 2021; Wainwright et al., 2021). Among them, enhancing the effectiveness of conventional antibiotics has been reported as a viable approach, and recent studies have extensively documented different categories of metabolites, as well as adjuncts for augmenting antibiotic potency (Liu et al., 2019; Lv et al., 2022). Certain compounds, including n-butanol and cajaninstilbene acid derivatives (Liu et al., 2019; Lv et al., 2022), have demonstrated therapeutic efficacy against persistent bacteria. Nevertheless, these compounds could cause cellular toxicity. Facilitating persister resuscitation is also considered an effective treatment against bacterial persistence (Defraine et al., 2018). Specific signaling molecules involved in nutritional (Dunny and Leonard, 1997) and fatty acid signaling (Marques et al., 2014) can modulate bacterial metabolism, facilitating persister resuscitation. For example, Davies and Marques (2009) demonstrated that cis-DA, a fatty acid signaling molecule, could induce the dissipation of already-formed biofilms and inhibit biofilm development. Marques et al. (2014) reported that cis-DA could induce a bacterial transition from a persistent to a highly antimicrobial-sensitive state. Compared to those treated with ciprofloxacin alone, the presence of cis-DA resulted in a significant reduction of 1 to 2 logarithmic orders in the population of persister cells for both *P. aeruginosa* and *E. coli* (Marques et al., 2014). Similarly, our findings demonstrated that cCF10, existing as short peptides in *Enterococci*, regulated the formation of *E. faecalis* persister cells in a concentration-dependent manner. The persister rate decreased from 0.109 to 0.050 and 0.047% when *E. faecalis* OGIRF was exposed to cCF10 concentrations of 10 and 12 ng/mL (Figure 1A).

Interestingly, we observed a gradual increase in the persister rate of *E. faecalis* during growth, which was accompanied by an upward trend in extracellular cCF10 pheromone concentration. This phenomenon could be attributed to the fact that the extracellular pheromone concentrations ranged from 0.04 to 0.08 ng/mL (Figure 2), whereas the inhibitory effects against *E. faecalis* persistence were observed when the additional cCF10 concentration reached 10 ng/mL (Figure 1), indicating that bacterial persistence could only be suppressed at a specific pheromone concentration threshold. Similarly, in our previous studies on conjugative transfer of plasmid, we also found that cCF10 induced the active state of bacteria in a certain range and then induced the transfer of plasmid, whereas higher concentrations of cCF10 inhibited the process (Yang et al., 2022). It may be because the high concentration of cCF10 affects the normal metabolism of bacteria. Besides, our results also indicated that the growth of OG1RF was inhibited by 40 ng/mL of cCF10 (Supplementary Figure S6). Concentrations of cCF10 below 40 ng/mL above 10 ng/mL affected OG1RF metabolism, although not OG1RF growth. Therefore, it is that elevated cCF10 levels disrupt bacterial metabolism and lead to bacterial persistence. Our findings also confirmed that biofilm content increased gradually during the growth process and that the gradual augmentation of biofilm content increased the persistence rates (Perez et al., 2014). However, elevated levels of cCF10 alone could not reverse this phenomenon. Consequently, we elucidated the two modes that impact the persistence process of *E. faecalis*, specifically the regulation of biofilm formation and pheromone effects. This deduction offers valuable insights into future efforts to combat *E. faecalis* persisters.

Subsequently, we investigated the mechanism by which cCF10 regulates the formation of persisters. Our results confirmed that the Opp system was suppressed during persistence, indicating a partial closure of the pheromone intake pathway in *E. faecalis*. Moreover, the results further confirmed that cCF10 modulated the expression of Opp2 system (Figure 4), implying that cCF10 may induce the Opp2 system. Peptide uptake in numerous bacterial species is predominantly facilitated by oligopeptide permease systems, which consists of a set of five protein constituents (Dunny, 2013). The OppA lipoprotein is a well-conserved protein known for its ability to bind peptides. It interacts with an ABC transporter system consisting of two channel-forming proteins (OppB and OppC) and two membrane-associated ATPases (OppD and OppF; Berntsson et al., 2012; Moraes et al., 2014). Together, this system facilitates the binding and transport of a wide range of peptides across different species (Moraes et al., 2014). Exogenous pheromone cCF10 is transported into the cell via the Opp system during ATP hydrolysis (Berntsson et al., 2012; Dunny, 2013). Segawa et al. (2021) indicated that the pheromone import in *E. faecalis* OG1RF was dependent on a functional Opp2 system. The Opp transport protein system, besides serving as a signaling molecule for intercellular communication in Gram-positive bacteria, also can capture extracellular nutrients (Moraes et al., 2014). Therefore, we thought that activation of the Opp system by cCF10 sustained cellular energy metabolism, thus inhibiting bacterial persistence. Formation of the persistence is induced by growth stagnation (Manuse et al., 2021). Bekale et al. (2023) reported that the persistence of *E. coli* might be regulated by ATP levels. Notably, accumulation of (p)ppGpp stimulated the production of toxin, thereby causing membrane potential collapse and ATP depletion, leading to bacterial dormancy (Pan et al., 2023). Analysis of the transcriptome results showed that cCF10 affected the expression of energy metabolism and (p)ppGpp related genes in *E. faecalis* (Figure 3). Two enzymes RelA (synthase) and SpoT (hydrolase) maintain a steady state of (p)ppGpp (Hobbs and Boraston, 2019). Simultaneously, inactivation of *phoU* can enhance (p)ppGpp accumulation (Shang et al., 2020). This study revealed that exposure to cCF10 upregulated the expression of *phoU*, thereby inhibiting (p)ppGpp accumulation (Figure 5). Further, accumulation of (p)ppGpp inhibited bacterial energy metabolism, thereby inducing bacterial dormancy (Pan et al., 2023). Our results demonstrated that cCF10 promoted the expression of *atpB* and *atpD* (Figure 6), which encode ATP synthetases.

Therefore, the present results indicate that the exogenous cCF10 entered the cell through the Opp channel, promoting ATP synthesis and sustaining bacterial energy metabolism. In general, the diminution of energy metabolism levels in bacteria induces bacterial persistence, as the majority of antibiotics exert their bactericidal effects by disrupting active, energy-dependent targets. In addition, the primary mechanism of action of the LVF employed in our study entails the suppression of bacterial DNA gyrase activity. Our study further confirmed that the external pheromone cCF10 enhanced the expression of *dnaE* (Figure 6), which encodes DNA polymerase III. This enzyme regulates the DNA replication by facilitating accurate and swift synthesis of DNA strands. The results suggested that cCF10 enhanced DNA replication, thereby augmenting the susceptibility of *E. faecalis* to the bactericidal effect of LVF. Consequently, cCF10 induced Opp system and entered bacterial cells to inhibit (p)ppGpp accumulation which maintained the metabolically active state of bacteria and reduced the generation of persister cells.

## 5 Conclusion

In conclusion, this study found that during the growth of *E. faecalis* OG1RF, accumulation of biofilm contributed to the development of antibiotic persistence. Moreover, we found for the first time that cCF10 prevented the formation of persister cells at certain concentrations. Surprisingly, cCF10 mediated the antibiotic persistence of *E. faecalis* OG1RF by altering the metabolic activity rather than inhibiting biofilm formation. Addition of cCF10 improved the Opp system and entered bacterial cells to suppress the accumulation of (p)ppGpp. This maintained the metabolically active state of bacteria and reduced the formation of persister cells. These results provide valuable insights and expand our understanding of the formation and control mechanism of persisters in *E. faecalis*.

## Data availability statement

The datasets presented in this study can be found in online repositories. The names of the repository/repositories and accession number(s) can be found in the article/Supplementary material.

## Author contributions

LZ: Writing – original draft, Writing – review & editing, Investigation, Formal analysis. XY: Writing – review & editing, Writing – original draft. XF: Formal analysis, Writing – review & editing. PY: Formal analysis, Writing – review & editing. XL: Formal analysis, Writing – review & editing. FW: Formal analysis, Writing – review & editing. ZS: Resources, Writing – review & editing. JW: Resources, Writing – review & editing. FS: Supervision, Writing – review & editing, Resources. ZQ: Supervision, Writing – review & editing, Resources.
