# Supplementary material for: Pheromone cCF10 inhibits the antibiotic persistence of Enterococcus faecalis by modulating energy metabolism
Source: Front Microbiol. 2024 Jul 8;15:1408701. doi: 10.3389/fmicb.2024.1408701 (PMC11260814; doi:10.3389/fmicb.2024.1408701)
Supplement: Supplementary file 1 [file Data_Sheet_1.docx]

Pheromone cCF10 inhibits the antibiotic persistence of Enterococcus faecalis by modulating energy metabolism

Li Zhu^1,2^ ^†^, Xiaobo Yang^2^ ^†^, Xinyue Fu^2,3^, Panpan Yang^2,4^, Xiaoli Lin^2,5^, Feng Wang^2,3^, Zhiqiang Shen^2^, Jingfeng Wang^2^, Feilong Sun^1*^, Zhigang Qiu^2*^

^1^ School of Environmental and Chemical Engineering, Xi’an Polytechnic University, Xian, China

^2^ Key Laboratory of Risk Assessment and Control for Environment and Food Safety, Tianjin Institute of Environmental and Operational Medicine, Tianjin, China

^3^ College of Oceanography and Ecological Science, Shanghai Ocean University, Shanghai, China

^4^ School of Public Health, North China University of Science and Technology, Tangshan, China

^5^ Key Laboratory of Karst Geological Resources and Environment, Guizhou University, Guizhou, China

^†^These authors contributed equally to this work and share first authorship

*** Correspondence:**Feilong Sun,
[sunfeilong@xpu.edu.cn](mailto:sunfeilong@xpu.edu.cn)
Zhigang Qiu,
[zhigangqiu99@gmail.com](mailto:zhigangqiu99@gmail.com)

# Supplementary Texts

**Text S1** **Construction of mutant strain OG1RFΔ*ccfA***

The genomic DNA was isolated from OG1RF using a commercially available DNA extraction kit following the manufacturer's instructions. The *ccfA* gene was amplified using the *ccfA*-F/*ccfA*-R primer pair, and the 880 bp PCR product was digested with BamHI/EcoRI and ligated into the similarly digested integration vector pk18mobsacB (YouBio, China) with pEASY-Uni Seamless Cloning and Assembly Kit (TRANSGEN, Beijing, China). The recombinant vector pk18-*ccfA* was transformed into *Escherichia coli* DH5α and spread on the LB medium. The plasmid pk18-*ccfA* was extracted from kanamycin (Km, 80 mg/L, Sangon, Biotech, China)- resistant colonies and verified by double digestion and DNA sequencing. The suicide vector pk18-*ccfA* was electroporated into OG1RF-competent cells, and the recombinant strains OG1RFΔ*ccfA* were selected by Km-resistance and sucrose-sensitivity plates.

**Text S2 Measurement of MIC and bactericidal experiment**

Antibiotics were added separately to cultures at the followings: levofloxacin hydrochlorid (Lvf), tetracyclin (Tet), and vancomycin (Van) in order to establish a screening model of the *E. faecalis* persisters. The overnight cultured OG1RF was diluted to 10^5^ CFU/mL with BHI, and then antibiotics with a concentration range of 0-256 mg/L were added separately using a two-fold gradient dilution method. The mixed culture was then added to a 100-well plate and placed in an Automatic Growth Curve Analyzer (Bioscreen C, Finland) to measure the OD_600_, and the time of the measurement was set at 12 h. MIC measurement was used for determination of the proper working concentration to persisters and recheck the absence of resistant OG1RF after antimicrobial exposure.

Thereafter, antibiotics at a concentration of 10 × MIC were added to the cultures separately to the OG1RF cultures in the logarithmic growth phase and counted the number of bacteria before and eight hours after the addition of antibiotics in order to compare the bactericidal effect of different antibiotics. A portion of the sample is removed and diluted at the designated time points and the appropriate dilutions of the samples were then plated on BHI agar for counting the surviving bacteria (CFU/mL).

**Text S3 The effects of cCF10 on the growth in *E. faecalis***

We investigated the effect of exogenous pheromone cCF10 exposure on the growth of OG1RF in order to determine the cCF10 concentrations in subsequent experiments. The overnight cultured OG1RF was diluted to 10^5^ CFU/mL in BHI. Then different concentrations of pheromones cCF10 (0.1 ng/mL, 0.5 ng/mL, 1 ng/mL, 5 ng/mL, 10 ng/mL, 20 ng/mL and 40 ng/mL) were added separately to the bacteria cultures. The mixed cultures were then added to a 100-well plate and placed in an Automatic Growth Curve Analyzer (Bioscreen C, Finland) to measure the OD_600_.

**Text S4 Detection of the extracellular pheromone cCF10**

The LC-MS analysis was conducted using the Ultimate 3000 UHPLC-Q Exactive instrument (Thermo Scientific, USA). Chromatographic separation was performed using an Eclipse Plus C18 column (4.6 × 100 mm, 5 µm). The mobile phase consisted of solvent A (acetonitrile) and solvent B [0.1% formic acid (F809712 Macklin, Shanghai, China) and 5 mM ammonium acetate (A801012 Macklin, Shanghai, China) in water]. The column was heated at 40℃. The flow rate and sample volume were 0.5 mL/min and 50 μL, respectively. The gradient elution was performed as follows. The process started with 90% of solvent A. The concentration of solvent A was decreased linearly from 90% to 20% from 1 to 11 min and retained at 20% for 5 min. The concentration of solvent A was increased linearly from 20% to 90% from 15 to 15.1 min and retained at 90% till the end. The specific mass spectrometry conditions are presented in Table S2.

**Text S5 Transcriptome sequencing and data analysis**

RNA isolation and qualification: RNA was extracted using the TRIzol method (Invitrogen, CA, USA) and treated with RNase-free DNase I (Takara, Kusatsu, Japan). RNA degradation and contamination was monitored on 1% agarose gels. RNA was quantified using Agilent 2100 Bioanalyzer (Agilent Technologies, CA, USA), the quality and integrity were assessed by NanoDrop spectrophotometer (Thermo Scientific, DE, USA).

Library preparation for Transcriptome sequencing: A total amount of 3 μg RNA per sample was used as input material for the RNA sample preparations. Use Vazyme Ribo-off rRNA depletion kit (bacteria) (Vazyme, Nanjing, China) to remove rRNA. Sequencing libraries were generated using NEBNext UltraTM RNA library Prep Kit (NEB, USA). Following purification, the RNA is fragmented into small pieces using divalent cations under elevated temperature. The cleaved RNA fragments are copied into first strand cDNA using reverse transcriptase and random primers. Strand specificity is achieved by replacing dTTP with dUTP in the Second Strand Marking Mix (SMM), followed by second strand cDNA synthesis using DNA Polymerase I and RNase H. The incorporation of dUTP in second strand synthesis quenches the second strand during amplification, because the polymerase used in the assay is not incorporated past this nucleotide. The addition of Actinomycin D to First Stand Synthesis Act D mix (FSA) prevents spurious DNA-dependent synthesis, while allowing RNA-dependent synthesis, improving strand specificity. These cDNA fragments then have the addition of a single 'A' base and subsequent ligation of the adapter. The products are then purified and enriched with PCR to create the final cDNA library. Library quality was assessed on the Agilent Bioanalyzer 2100 system. The library preparations were sequenced on an Illumina Novaseq 6000 platform by Beijing Allwegene Technology Company Limited (Beijing, China) and paired-end 150bp reads were generated.

Quality control: Raw data (raw reads) of fastq format were firstly processed through in-house perl scripts. In this step, clean data (clean reads) were obtained by removing reads containing adapter, reads containing ploy-N (N>10%) and low-quality reads (Q<5) greater than 50% from raw data. At the same time, Q20, Q30 and GC content of the clean data were calculated. All the downstream analyses were based on the clean data with high quality.

Reads mapping to the reference genome: The adaptor sequences and low-quality sequence reads were removed from the data sets. Raw sequences were transformed into clean reads after data processing. These clean reads were then mapped to the reference genome sequence by Bowtie2 v2.2.6. Only reads with a perfect match or one mismatch were further analyzed and annotated based on the reference genome.

Novel transcripts prediction: The Bowtie2 mapping results were assembled with Rockhopper software and compared with the annotated gene models to find new transcript regions. The Blastx program is compared with the NR library (the evalue is set to le-5), and the newly predicted transcript region is annotated, and the annotated transcript region is regarded as a new transcript region with coding potential.

SNP / InDel analysis: Picard-tools v1.96 and samtools v0.1.18 were used to sort, mark duplicated reads and reorder the bam alignment results of each sample. GATK3 software was used to perform SNP and InDel calling.

Gene structure analysis: In prokaryotic genomes, functionally related genes are usually clustered and regulated by a single upstream promoter and a single downstream terminator. Such genetic structure is called operon. The genes in the same operon are transcribed together into an mRNA strand and then translated to different proteins. According to the loactions of reads in the reference genome, transcription start sites (TSS) and transcription termination sites (TTS) of operons are predicted using Rockhopper. Then promotors prediction were applied using 700-bp sequences in the upstream of TSS, by time-delay neural network (TDNN) method. For 5' UTR sequences, SD sequences were predicted using RBSfinder (rbs region length = 50). For 3' UTR sequences, ρ-independent terminators were predicted using TransTermHP. Accoding to orientations in the genome, Cis-NATs are clasified to three types: enclosed (full overlaped by the sense transcript), convergent (3'-3' overlap) and divergent (5'-5' overlap). The genomic loci, type and numbers of cis-NATs were indentified using the strand- specific RNA-seq data.

sRNA Analysis: In prokaryrotes, non-coding RNAs with length between 50 and 500 nt are defined as small RNA (sRNA). Novel intergenic transcripts were discovered by Rockhopper, and alignned with sequences in NCBI NR database using Blastx. Novel transcripts without NR annotation were sRNA candidates. RNAfold and IntaRNA were applied to predict the secondary structures and targeted genes of sRNAs, respectively.

Quantification of gene expression level: HTSeq v 0.5.4 p3 was used to count the reads numbers mapped to each gene. Gene expression levels were estimated by fragments per kilobase of transcript per million fragments mapped (FPKM).

Differential expression analysis: Differential expression analysis of two conditions/groups (two biological replicates per condition) was performed using the DESeq R package (1.10.1). DESeq provide statistical routines for determining differential expression in digital gene expression data using a model based on the negative binomial distribution. The resulting *P*-values were adjusted using the Benjamini and Hochberg’s approach for controlling the false discovery rate. Genes with an adjusted *P*-value <0.05 found by DESeq were assigned as differentially expressed.

Kyoto Encyclopedia of Genes and Genomes (KEGG) enrichment analysis of differentially expressed genes: KEGG is a database resource for understanding high-level functions and utilities of the biological system, such as the cell, the organism and the ecosystem, from molecular-level information, especially large-scale molecular datasets generated by genome sequencing and other high-through put experimental technologies. We used KOBAS software to test the statistical enrichment of differential expression genes in KEGG pathways.

**Text S6 Measurement of Extracellular polymers (EPS)**

The EPS was extracted by ultrasonication. 10 mL of bacterial culture medium was taken and centrifuged at 6000 rpm and 4℃ for 5 min, and then washed and resuspended with PBS twice to obtain 15 mL of bacterial suspension. The bacterial suspension was ultrasonicated at 20 kHz, 40 W, and ice bath for 15 min, then centrifuged at 15000 rpm for 30 min, and the supernatant was filtered through 0.22 μm membrane to obtain the EPS. The polysaccharide content of EPS was determined by the Total Polysaccharide Content Assay Kit (Isetsu Bio-tech Co., Ltd., Lianyungang, China), and the protein of EPS was determined by the BCA Protein Concentration Assay Kit (Biyuntian Bio-Tech Co., Ltd., Shanghai, China). The protein content of EPS was determined by BCA Protein Concentration Kit (Biyuntian Biotechnology Co., Ltd., Shanghai, China), and the DNA content of EPS was determined by Nano drop (Thermo Fisher Scientific).

# Supplementary Figures and Tables

## Supplementary Figures


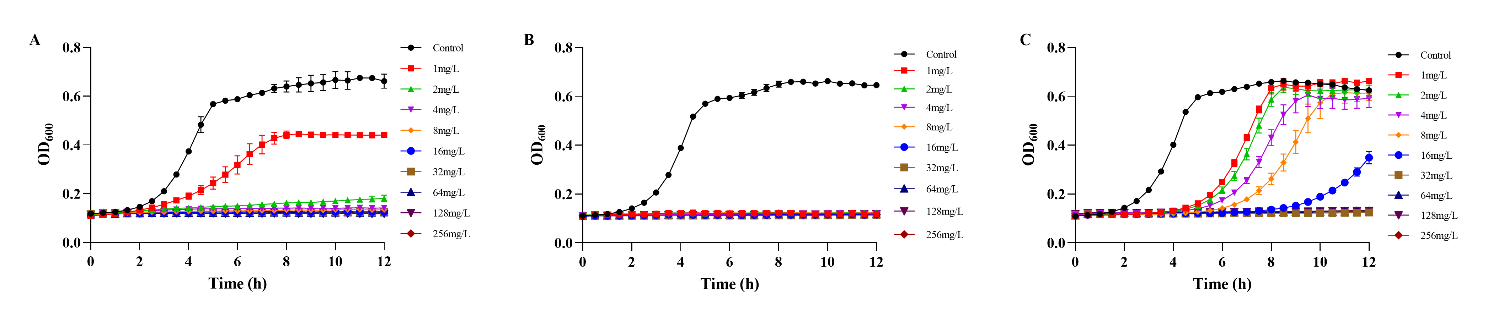


**Supplementary Figure 1.** MIC of different antibiotics in *E. faecalis.* MICs of Lvf (A), Amp (B) and, Tet (C) in OG1RF were 2 mg/L, 1 mg/L and 16 mg/L, respectively. The results represent the mean ± standard deviation (SD) of three biological replicates.


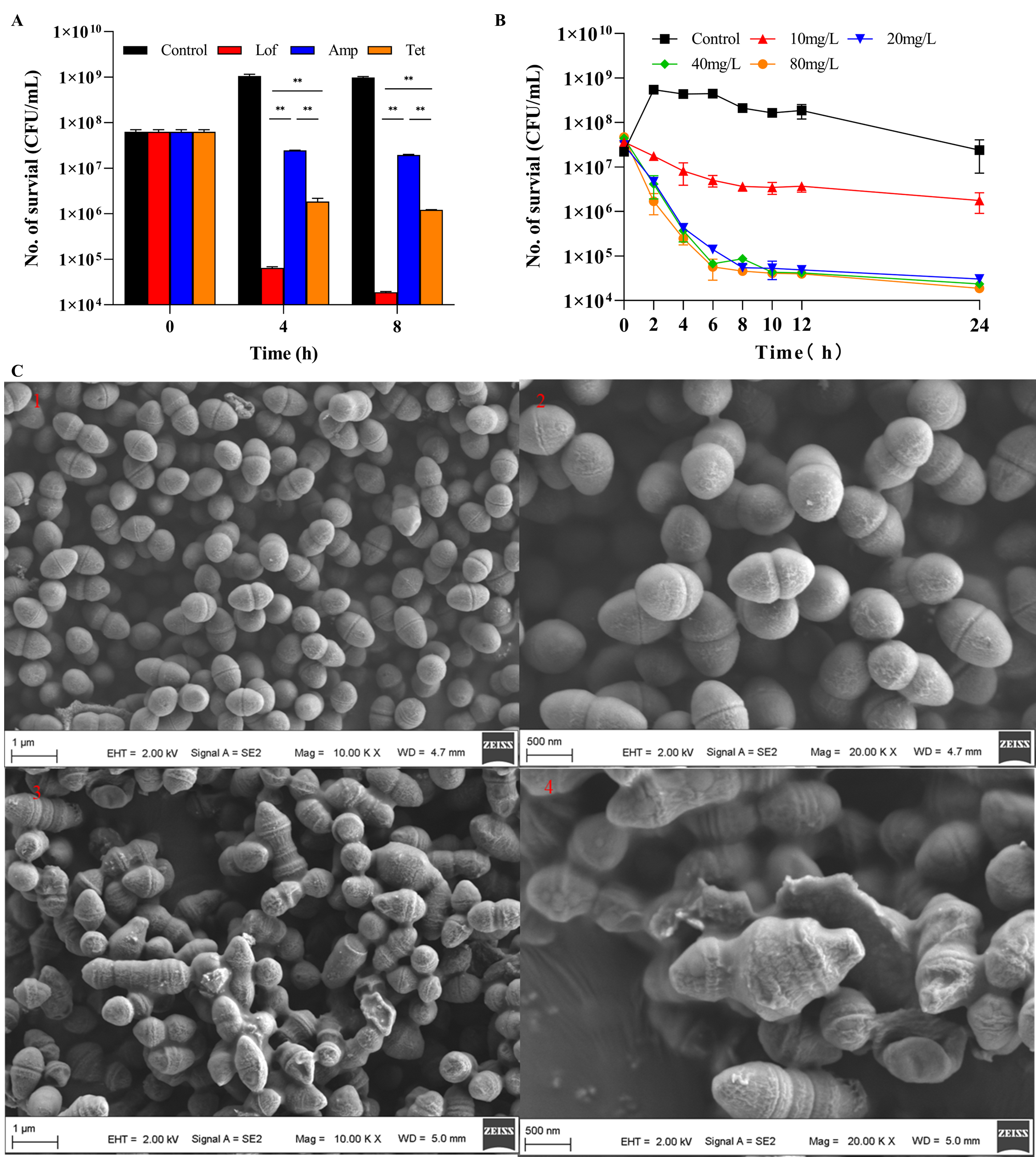


**Supplementary Figure 2.** Lvf screening for *E. faecalis* persisters*.* (A) Bactericidal effects of different antibiotics on OG1RF. Significant differences between groups were obtained using analysis of variance and marked with ***P* < 0.01. (B) Biphasic killing curve after Lvf exposure. The results represent the mean ± SD of three biological replicates. (C) The SEM images of *E. faecalis*. 1-2, the control group; 3-4, The morphology of bacteria after Lvf treatment.


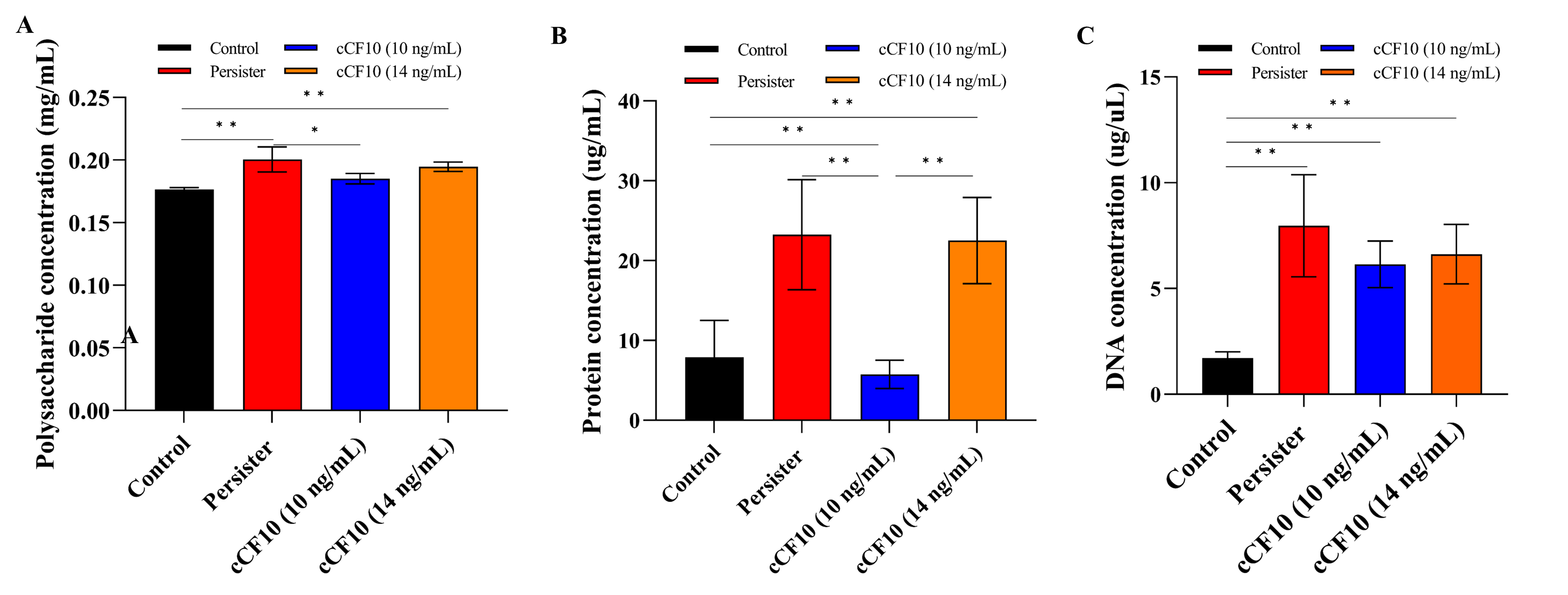


**Supplementary Figure 3.** Effect of cCF10 on modulating EPS formation during the persistence of *E. faecalis*. (A) polysaccharide (B) protein, and (C) DNA.


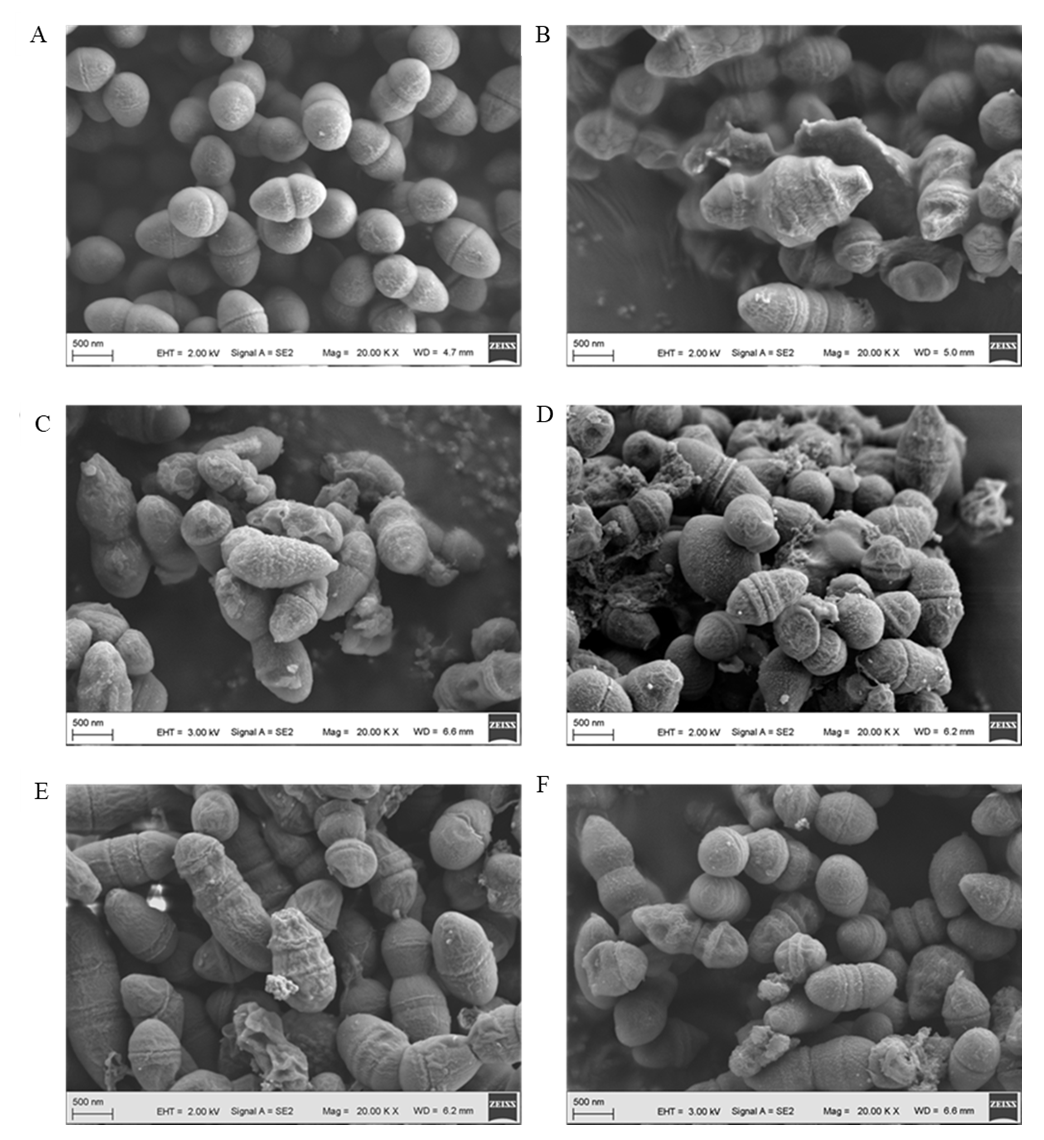


**Supplementary Figure 4.** The SEM images of resuscitation of OG1RF persisters (a): normal OG1RF; (b): OG1RF persisters; (c), (e): resuscitation of OG1RF persisters for 0.5h, 1h; (d), (f): resuscitation of OG1RF persisters exposed 10 ng/mL cCF10 for 0.5h, 1h.


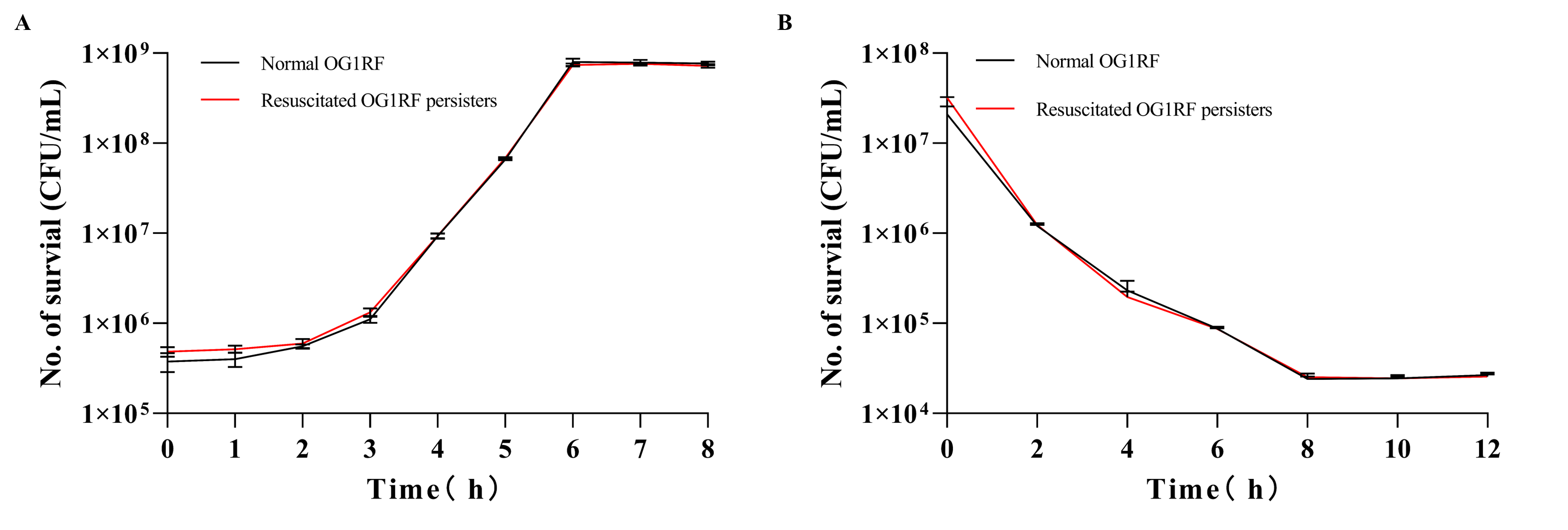


**Supplementary Figure 5.** OG1RF growth and sterilization curves. (A) OG1RF growth curves; (B): OG1RF sterilization curves.


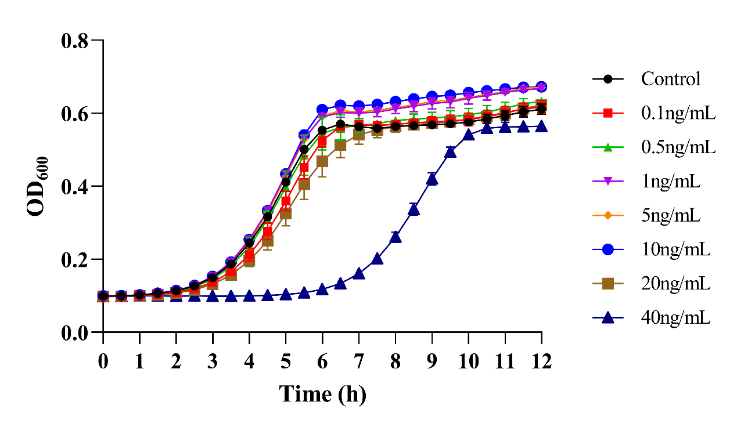


**Supplementary Figure 6.** Effects of cCF10 on the growth of *E. faecalis.* The results represent the mean ± SD of three biological replicates.


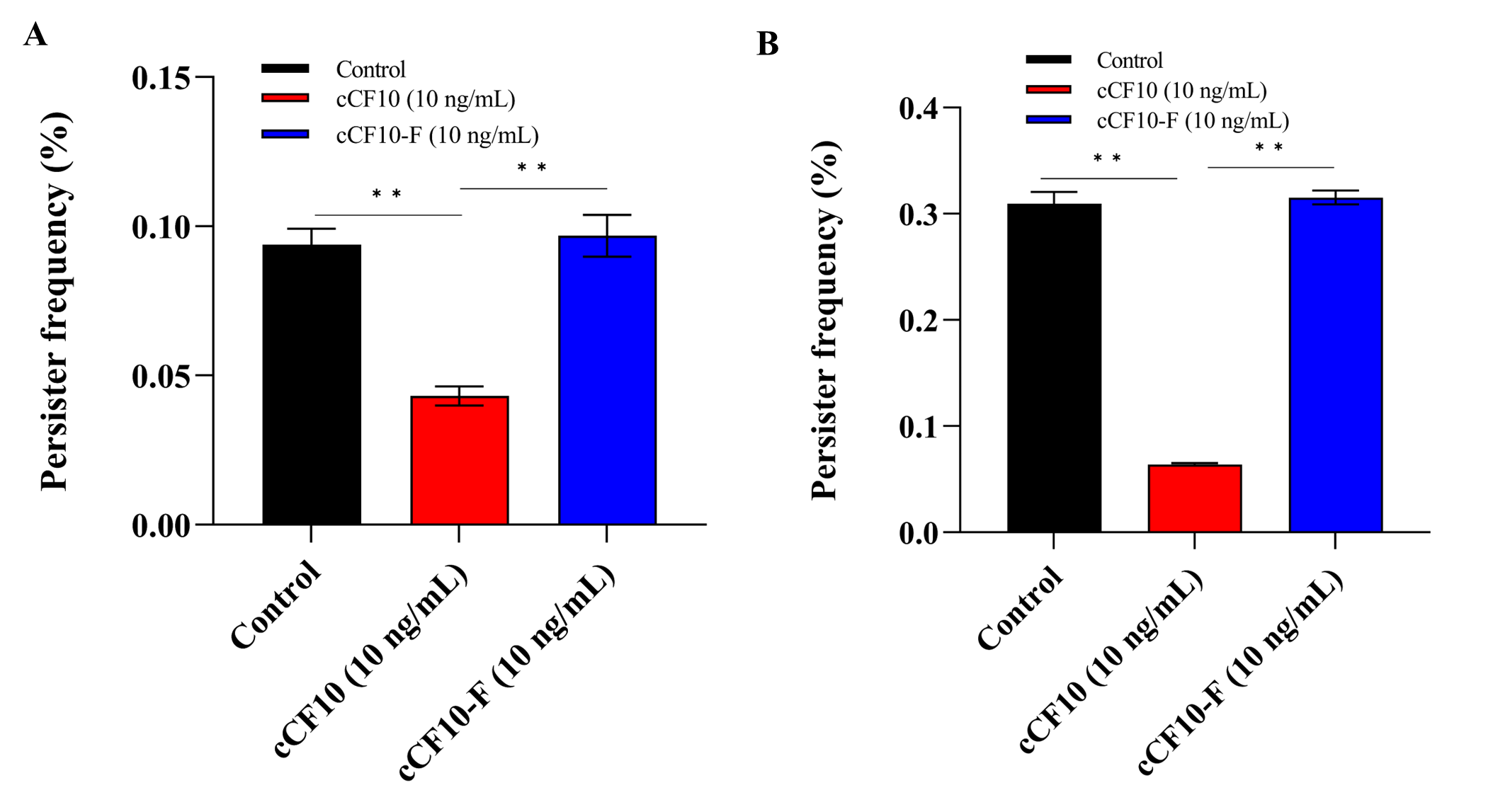


**Supplementary Figure 7.** The effect of pheromones on the formation of persisters of *E. faecalis*. (A) OG1RF, (B) OG1RF*∆ccfA*. Significant differences between groups were obtained using analysis of variance and marked with ***P* < 0.01. The results represent the mean ± SD of three biological replicates.


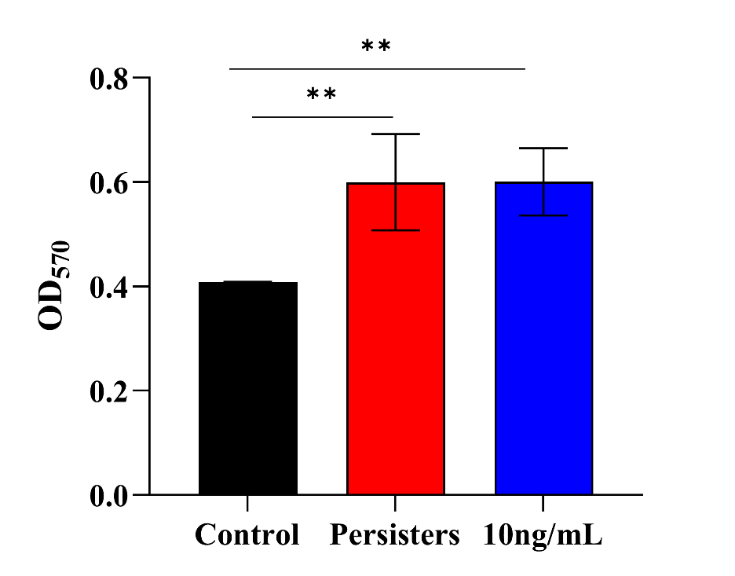


**Supplementary Figure 8.** Effect of cCF10 on modulating biofilm formation during the persistence of *E. faecalis*. Significant differences between groups were obtained using analysis of variance and marked with **P* < 0.05 and ***P* < 0.01. The results represent the mean ± SD of three biological replicates.


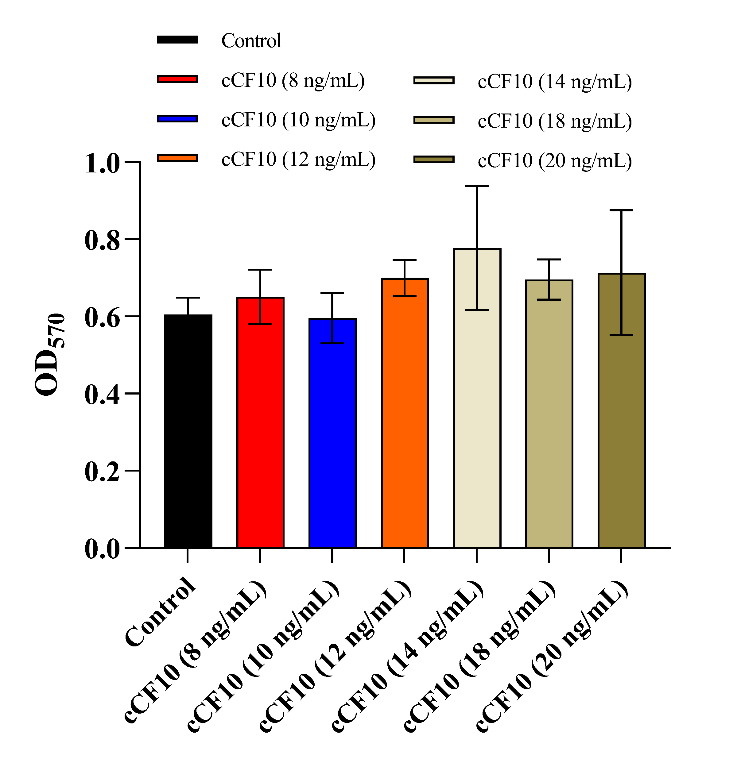


**Supplementary Figure 9.** Effect of different concentrations of cCF10 on OG1RF biofilm formation.

## Supplementary Tables

**Table S1. Sequence of primers for qRT-PCR analysis**

| Gene name | Primer Sequence (5’ to 3’) | | Length of the product (bp) | |  |
| --- | --- | --- | --- | --- | --- |
| *opp2A* | F: GGCGGTGTTTGGGGTTCAGT | | 249 | |  |
|  | R: TTTCGCTTTCATCGCTTCTACAAT | |  |  |  |
| *opp2B* | F: TTTGCCGTGCCATTATTCATCTTT | | 117 | |  |
|  | R: CGGCCACGTTCCCTCAGC | |  |  |  |
| *opp2C* | F: TATTATCGGGGTCGGTTTAG  R: CTCGGCGCCCTTCAGAT | | 236 | |  |
| *opp2D* | F: AAGCACGGGCCATTGAACTG  R: GACCCGCATACATAACCGCTACAC | | 309 | |  |
| *opp2F* | F: TTGGCGGTTGATGGTGTGGA  R: TCGACTTCGCGGCTTGGTTAC | | 171 | |  |
| *relA* | F: TGAACGGGCAATGCAGGAAGAGT  R: AACGCCACCGACGCCAATCACAAA | | 319 | |  |
| *spoT* | F: GCCGCATCGCCCGTGAAACTCT  R: TGGCCCAGCACGCGATAACAGGT | | 370 | |  |
| *phoU* | F: GGTTAGTAGTGCCGTCCAT  R: GGTCCGCCATTCTTTCTA | | 221 | |  |
| *danB* | | F: TCAAAGATCGCCTAGAACAAGAGA  R: AAGCCGGCAGTCATTTTATCCA | | 391 | |
| *danE* | | F: ATGGGGCGGTTGAATTTTATGAA  R: GAGCGCTGGATTACGTTGTTGATT | | 386 | |
| *recG* | | F: ATTTCGCCCCCATTTATCAT  R: TTTTTCCGCTTTTTCTTGTTTC | | 284 | |
| *atpB* | | F: AATGGCGTTTACCCTGTTCA  R: CTTCGCCGGCAAAAATGTT | | 311 | |
| *atpD* | | F: ACGCCGTGTTCAATTCTTCTTATC  R: GGCATCTTCAATTTTACCGACACT | | 178 | |
| 16s rRNA | | F: GCGGCGTGCCTAATACA  R: CCGCGGGTCCATC | | 219 | |

**Table S2. The mass spectrometry conditions**

| Parameter name | Mass spectrum condition |
| --- | --- |
| mass Spectrometer | Thermo Scientific Q Exactive |
| ion source | HESI |
| sheath gas rate (arb) | 40 |
| auxiliary gas rate (arb) | 10 |
| spray voltage (kV) | 3.0 (Positive ion) |
| capillary temperature (°C) | 320 |
| s-lens | 50% |
| scanning mode | PRM |
| parent ion (m/z) | 790.50730 |
| qualitative daughter ions (m/z) | 314.2074, 296.19690, 213.15993 and 409.28125 |
| quantitative ion selection (m/z) | 314.20743 |
| fragmentation energy | NCE 27 |
